# Biomimetic Nanoarchitectures for Light Harvesting: Self-Assembly of Pyropheophorbide-Peptide Conjugates

**DOI:** 10.1021/acs.jpclett.0c02138

**Published:** 2020-09-04

**Authors:** Elena Meneghin, Francesca Biscaglia, Andrea Volpato, Luca Bolzonello, Danilo Pedron, Elisa Frezza, Alberta Ferrarini, Marina Gobbo, Elisabetta Collini

**Affiliations:** † Department of Chemical Sciences, University of Padova, via Marzolo 1, 35131 Padova, Italy; ‡ Université de Paris, CiTCoM, CNRS, F-75006 Paris, France

## Abstract

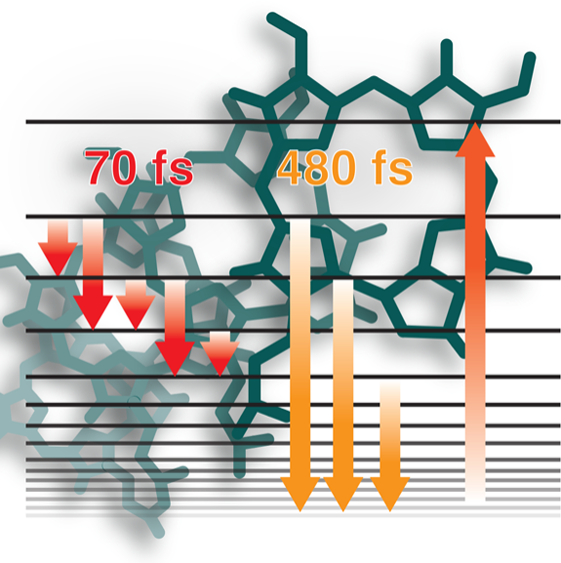

The biological light-harvesting process offers an unlimited source of inspiration. The high level of control, adaptation capability, and efficiency challenge humankind to create artificial biomimicking nanoarchitectures with the same performances to respond to our energy needs. Here, in the extensive search for design principles at the base of efficient artificial light harvesters, an approach based on self-assembly of pigment-peptide conjugates is proposed. The solvent-driven and controlled aggregation of the peptide moieties promotes the formation of a dense network of interacting pigments, giving rise to an excitonic network characterized by intense and spectrally wide absorption bands. The ultrafast dynamics of the nanosystems studied through two-dimensional electronic spectroscopy reveals that the excitation energy is funneled in an ultrafast time range (hundreds of femtoseconds) to a manifold of long-living dark states, thus suggesting the considerable potentiality of the systems as efficient harvesters.

Natural light-harvesting complexes (LHCs) are a fascinating machinery in which structure, dynamics, and functionality are intimately related. The light-harvesting action in LHCs is performed by purposefully organized chromophores, whose mutual interactions and couplings with the protein scaffold have been finely tuned by evolution to capture solar light with outstanding performances.^1^

Inspired by Nature, several methods have been explored in the past several decades for mimicking the efficiency of LHCs with artificial systems. Successful examples have been obtained by using hyperbranched polymers,^2,3^ functionalized nanostructures, and supramolecular assemblies,^3−9^ to cite just a few. The literature clearly shows that the field of bioinspired photosystems appears to be most promising but still in its infancy, and thus, an extensive number of efforts are ongoing.^10^ The use of functional soft materials designed through reversible bonding among structural units is an exciting possibility that is currently being intensively scrutinized. The abilities of the proposed materials to adapt and change in response to their environment, respond rapidly to external stimuli, be stable under illumination, and integrate many functionalities are all features that make these materials extremely interesting and promising.^11,12^

In this context, a particularly attractive approach for the construction of biomimetic nanoarchitectures is to exploit the self-assembly of suitably functionalized short amino acid sequences (peptides). Assemblies of bioinspired designed peptides exhibit remarkable functional behaviors. They can display structural and mechanical robustness yet can be controllably and reversibly disassembled. For this reason, the self-assembly of peptides has been revealed to be a powerful approach, and it is often used as a bottom-up strategy for the synthesis of nanomaterials with complex, hierarchical architectures.^13−16^

Here the same strategy is exploited to prepare biomimetic complexes for light harvesting, promoting the self-assembly of pigment-peptide conjugates. Like in natural light-harvesting systems, the pigments are responsible for light absorption, while the amino acid sequence has a structural and solubility function.

The energy migration within the manifold of the excitonic states formed upon self-assembly has been characterized in the ultrafast time regime by two-dimensional electronic spectroscopy (2DES), now recognized as one of the most advanced and powerful spectroscopic techniques for unveiling the finest details of the mechanism and dynamics of energy transport. We found that our nanoarchitectures fulfill some of the most critical requirements characterizing a good antenna, including the presence of long-living collector states at lower energies, to which the excitation energy is quickly funneled. The design and the preparation of the artificial antenna have been driven by some of the main design principles inspired by biological antennas.^17,18^

First, an efficient antenna must have strong and spectrally wide absorption bands in the visible range. As the absorbing chromophore, rather than the most widely used *meso-*tetraphenylporphyrin,^13,14,19,20^ pyropheophorbide *a* (PPh) is used. PPh is a free base derivative of a catabolite of chlorophyll *a*. PPh, like other molecules of the chlorines family, presents a higher oscillator strength in the so-called Q-bands with respect to other tetrapyrroles.^21^ Moreover, the *x* and *y* components of the Q bands, non-overlapping in PPh for symmetry reasons, together with the associated vibronic sidebands, give rise to a rich pattern of absorption bands in the range of 500–700 nm, which usually can be attained only mixing more than one chromophore.^22^ This is a particularly desired feature toward the realization of ideal panchromatic light harvesters.

In the studied conjugate, the PPh moiety is covalently linked to an artificial peptide H-Ala-(Aib-Ala)_7_-OH (*ap*) (Scheme 1 and the Supporting Information). In water, this sequence shows a strong propensity to maintain the same helical conformation as in organic solvents^23^ and to form supramolecular self-assembled structures.^24^ Here the self-assembly properties of the peptide in water are exploited to promote the controlled aggregation of the conjugate by solvent tuning.^25,26^

**Scheme 1:**
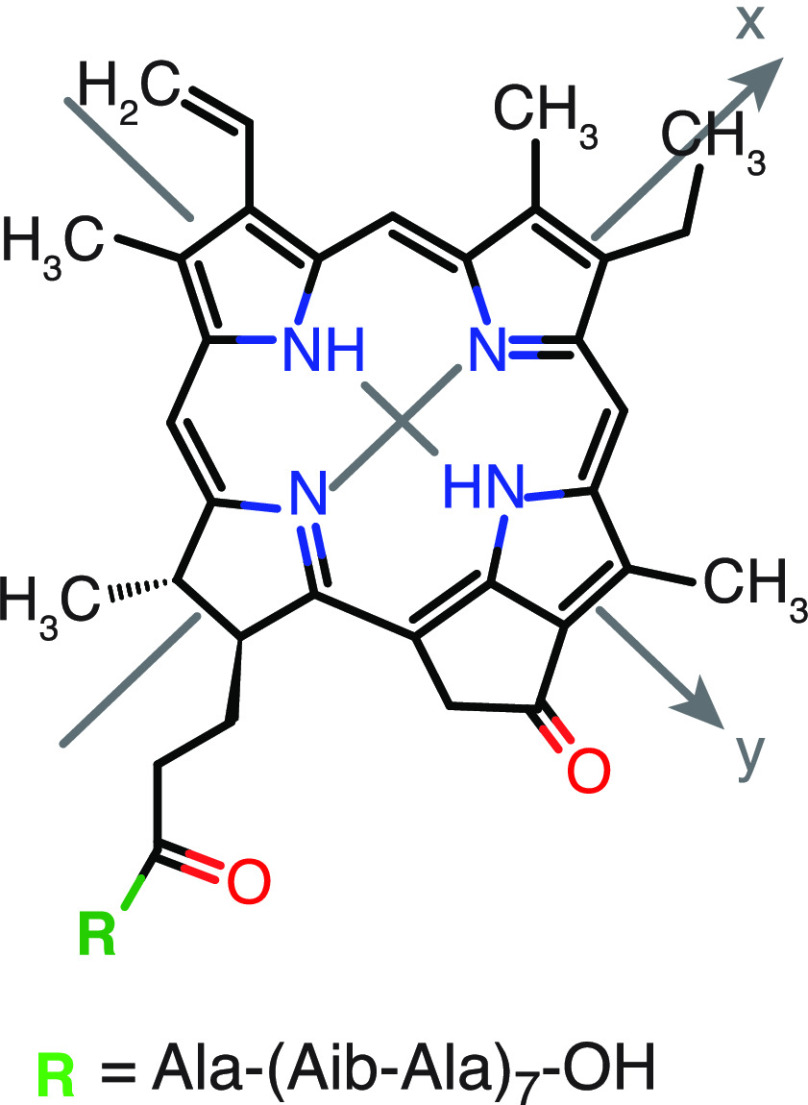
Molecular Structure of the PPh-*ap* Conjugate (Ala, alanine; Aib, 2-aminoisobutyric acid)

The self-assembly of the peptide moieties also constrains the organization of the hydrophobic PPh pigments attached as side chains,^14,16^ promoting, in this case, the formation of strongly coupled aggregates, as verified in the absorption and circular dichroism (CD) spectra (Figure 1). The formation of a dense network of interacting pigments, possibly giving rise to delocalized excitations and excitonic networks, is an important design principle in light harvesting.^8,9,20,27^ The formation of strongly interacting aggregates of chromophores is indeed a common strategy in Nature to shift and make broader the absorption spectrum of pigments.^28^ Bacteriochlorophyll aggregates in chlorosomes of green sulfur bacteria are an outstanding example of this strategy.^29^ Moreover, the generation of delocalized states is now recognized as a crucial criterion for improving the efficiency of light harvesting,^30−33^ and for promoting the generation of dark states at lower energies, which is useful for preventing radiative recombination.^31,34,35^

**Figure 1:**
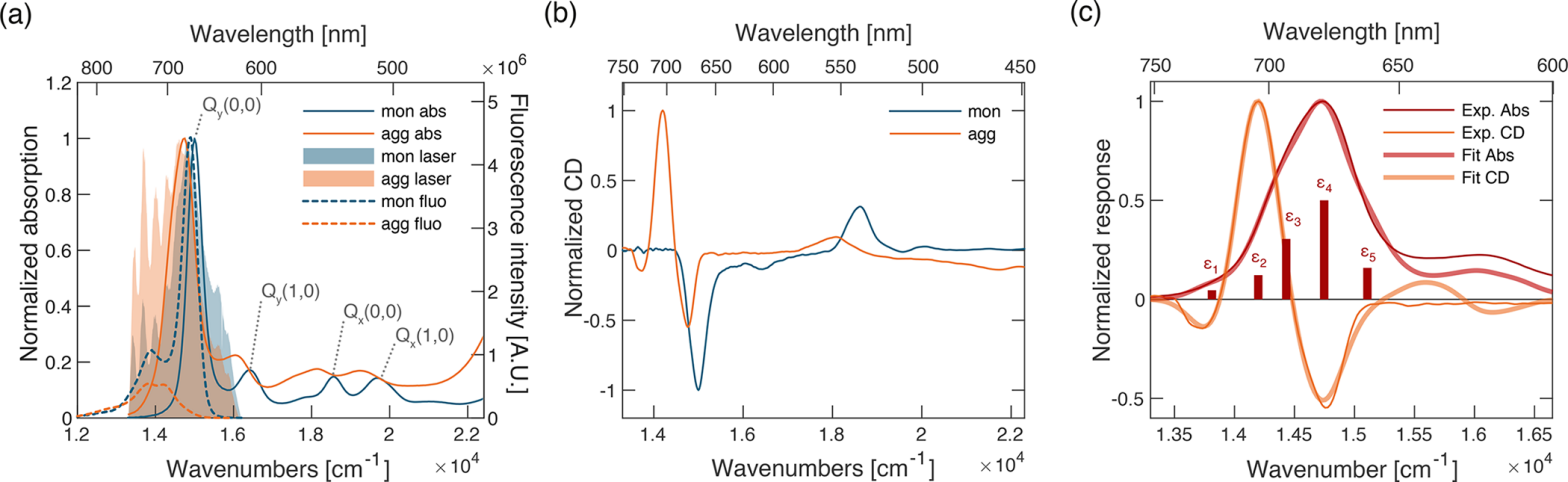
Linear characterization. (a) Normalized absorption spectra (solid lines) and emission spectra (dashed lines) of the monomeric (blue) and aggregated (orange) forms of the conjugate [monomer, solution in MeOH; aggregate, solution in a 1:9 (v/v) MeOH/H_2_O mixture]. Light blue and light orange areas represent the laser spectrum used for 2DES experiments on monomer and aggregate solutions, respectively. (b) CD spectra of the monomeric (blue) and aggregated (orange) conjugate. (c) Experimental (thin dark lines) and fitted (thick light lines) traces of the absorption (red) and CD (orange) spectra of the aggregate in the Q_*y*_ band region. The relative amplitude of the bright absorption transitions is reported as red bars.

The complex nature of the supramolecular assembly hampers a precise determination at the microscopic level of the pigments’ geometry and arrangement. Differently from other examples of self-assembly pigments reported in the literature where special functional groups were inserted to drive the self-assembly toward well-designed nanostructures,^14−16,36^ the conventional techniques of morphological characterization could not be successfully applied.

Although a precise characterization of the morphology of the self-assembled nanostructures is undoubtedly crucial to directly show the results of self-assembly, provide information about the self-assembly mechanism, and develop effective applications of the materials, here the main idea is to assess the photophysical characterization of the nanostructures and verify their dynamic behavior in the ultrafast regime to benchmark these systems as potential light-harvesting materials. It is well-known that, when it comes to excitonic systems, the electronic (and optical) properties are determined by the number of molecules over which the excitation is effectively delocalized rather than on their physical size.^37^ Therefore, a thorough morphological characterization of the nanoassemblies at this stage is not essential and will be the object of further investigations. The self-assembly is thus monitored by looking at the optical properties and comparing them with the results obtained for the monomeric non-aggregated forms.

In methanol (MeOH), the PPh-*ap* conjugate is stable in its monomeric form. When the solvent’s polarity is increased, for example, by adding water, the different hydrophilicity of the PPh and peptide moieties induces self-assembly and aggregate formation. We found that a 1:9 (v/v) mixture of MeOH and H_2_O is the best compromise for guaranteeing a good solubility of the conjugate and strong excitonic interactions among the pigments (see the Supporting Information). The proximity of chromophores in this self-assembled architecture induces excitonic interactions among them, manifested as specific spectral changes in the absorption, emission, and CD spectra.

Figure 1a compares the absorption spectra of the monomer and aggregate species in the Q-band region. A general broadening and red-shift of the bands are observed in the aggregate spectrum, in agreement with previous evidence for the formation of J-type aggregates of pheophorbide and chlorophyll-like derivatives.^38−40^ The presence of excitonic interaction is also confirmed by the inspection of the CD spectra in the visible region, illustrated in Figure 1(. While the monomeric PPh-*ap* conjugate presents the same intrinsic CD spectrum of the PPh molecule, the aggregate shows the typical dispersive shape due to excitonic interactions among pigments.^41,42^ The aggregation also promotes substantial changes in the fluorescence emission behavior of the conjugate (Figure 1a). While the monomer presents a spectrum with the typical mirror symmetry of tetrapyrrole compounds, the profile of the aggregate emission spectrum shows two red-shifted maxima. Moreover, the aggregate emission is strongly suppressed (the measured fluorescence quantum yield decreases from 41.3 ± 4.2% for the monomer to 6.4 ± 0.4% for the aggregate), suggesting the presence of effective nonradiative pathways involving weakly emissive lower-energy states, as suggested below.

To have a clearer view of the energy landscape promoted upon aggregation in the region of the low-energy Q_*y*_ bands, the absorption and CD spectra have been simultaneously analyzed with a global multicomponent fitting procedure (section S2.2). First, we simulated the absorption and CD spectra of the monomer. The spectra of the monomer in this spectral region could be excellently reproduced considering a single-transition model function coupled with six vibrational modes, whose frequency has been determined through independent resonant and nonresonant Raman measures (Supporting Information). The absorption and CD spectra of the aggregate have been fitted simultaneously by means of a global fitting procedure based on the variable projection algorithm, as described in ref (43). In this procedure, the transition frequencies have been treated as shared global parameters. Each transition in the aggregate has then been modeled using the same line shape function previously determined through the fitting of the monomer. The results of this global analysis are reported in Figure 1c and revealed the presence of at least five distinct transitions in the Q_*y*_ spectral region at 13810, 14200, 14430, 14750, and 15110 cm^–1^. The identified excited states have been labeled as ε*_n_*, with *n* ranging from 1 to 5. Note that the two lowest-energy states, ε_1_ and ε_2_, are characterized by considerably small transition dipole moments, and they can be considered as partially forbidden states. Their energies match the position of the maxima recorded in the experimental fluorescence spectrum, and their marked dark character also justifies the reduced quantum yield of the aggregate. Note that the presence of these (almost) dark states on the low-energy tail of the Q_*y*_ bands is particularly promising from the perspective of preparing an artificial antenna because it achieves the requirement of having low-energy levels acting as final excitation collectors.

The lifetime of the emitting states has been determined through time-resolved fluorescence decay measurements in a time-correlated single-photon counting (TCSPC) apparatus. This experiment (results shown in the Supporting Information) revealed that, despite the expected overall shortening of the lifetime of the aggregate with respect to that of the monomer, the emission dynamics remain in the nanosecond regime, confirming the potentiality of ε_1_ and ε_2_ as “energy storage” states.

To gain insight into the fundamental interactions leading to the formation and stabilization of aggregates, we have performed all-atom simulations (ATMD)^44^ of single pigment-peptide conjugates (monomers) and pairs of these (dimers) in MeOH and in water (Figure 2 and the Supporting Information). Although it is likely that in solution aggregates of different sizes may form, dimeric units can be reasonably considered as the basic elements for the interpretation of the system’s photophysics and represent the ideal starting point for investigating the fundamental interactions leading to the stabilization of the supramolecular assemblies.

**Figure 2:**
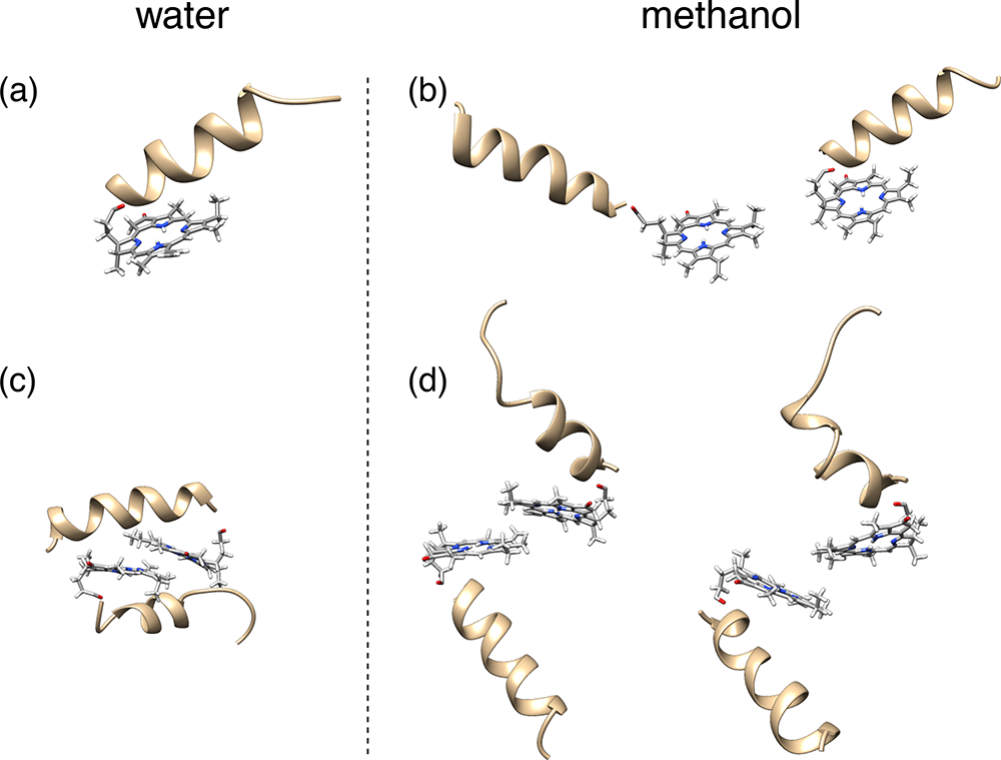
Molecular dynamics simulations. Snapshots from ATMD simulations of (a and b) a monomer and (c and d) a dimer of the PPh-*ap* aggregate in water and methanol. In water, both the monomer and the dimer exhibit small fluctuations around structures such as those shown here. In methanol, instead, there are larger fluctuations; the figure shows representative structures of the monomer and the dimer, as obtained from a clustering analysis of ATMD trajectories.

Meaningful differences appear already at the level of a monomer. The peptide chain in both solvents takes an α-helical conformation, in agreement with the experimental findings (see Figure S2a and refs (23) and (24)). Methanol appears to be a good solvent, where the chain moves freely and explores several relative orientations with respect to PPh. Two snapshots are shown in Figure 2b (see also Figures S10–S14 for further details). In water, on the contrary, the peptide sits on the plane of PPh and exhibits restricted mobility (Figure 2a); in this way, the conjugate takes a compact shape, which minimizes the contacts with the solvent. For dimers, guided by the experimental absorption spectrum, which suggests the presence of J-type aggregates, we have investigated structures with staggered PPh moieties at various angles with respect to each other, in the presence of geometric restraints that avoid the stacking of pigments into H-structures. Again, a distinct behavior is observed in water and in MeOH, which highlights the crucial role of the peptide in promoting the self-assembly of the dye moieties. In water, the peptide chain acts as a glue, which sticks the PPh moieties together. Starting from the conjugates at a certain distance, each peptide tends to get closer to the PPh in the other conjugate along the trajectory, and simultaneously also the two PPh moieties move closer (see Figures S13 and S14). Globular aggregates are formed, where the α-helices are well accommodated around the pigments. In MeOH, the peptide chains show high mobility with respect to the pigments, preventing the formation of stable aggregates. The different behavior in the two environments is illustrated in panels c and d of Figure 2, which show snapshots from trajectories of dimers in water and MeOH, respectively, started from the same initial configuration. These snapshots were selected on the basis of a clustering procedure,^44−47^ whereby geometrically similar structures are grouped into the same cluster (see section S3.2 for more details). In water, both for the monomer and for the dimer, most of the structures belong to the same cluster, whereas in MeOH, there is a larger variety of allowed geometries, which can be collected in a number of different clusters.

The study of the ultrafast dynamics in the Q_*y*_ spectral region from higher- to lower-energy states for the monomer and the aggregate has been performed by exploiting the 2DES technique in the photon echo BOXCARS configuration. Details of the experimental setup are provided in the Supporting Information and ref (48). 2DES is one of the most powerful techniques for characterizing the energetics and dynamics of energy transfer within multichromophoric systems.^27,49−55^ It is also particularly informative for the characterization of dark states.^52,56^ In this context, it appears to be the ideal technique for investigating the crucial role of (almost) dark states in the relaxation dynamics of the PPh-*ap* aggregate.

Figure 3 summarizes the results obtained for the PPh-*ap* monomer in MeOH. The dynamics of the PPh-*ap* aggregate closely resemble what has already been found for other isolated tetrapyrrole pigments in solution.^57−62^ The signal is dominated by a positive diagonal peak centered at ∼15000 cm^–1^, easily attributed to ground state bleaching (GSB) and stimulated emission (SE) of the resonant Q_*y*_ transition.

**Figure 3:**
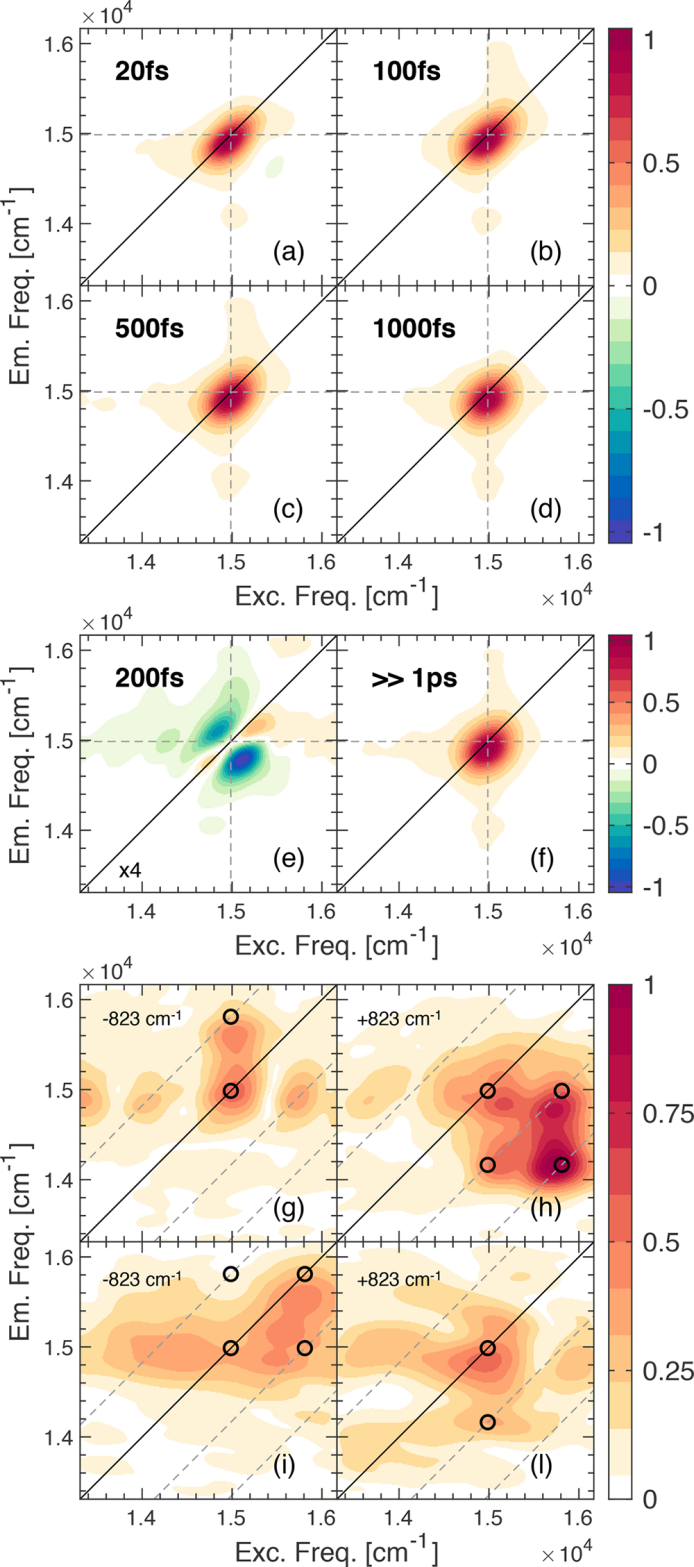
2DES characterization of the PPh-*ap* monomer in MeOH. (a–d) Pure absorptive 2DES maps of the monomer at selected values of population time *t*_2_. Dashed lines pinpoint the position of the Q_*y*_(0,0) transition. (e and f) 2D-DAS retrieved from the global fitting analysis of the PPh-*ap* aggregate in the MeOH data set. (g–l) Fourier maps at ω_2_ = 823 cm^–1^ obtained from the (g and h) rephasing and (i–l) non-rephasing signal. Black dots pinpoint coordinates where vibrational modes are expected to contribute.^57,68^

In the time evolution of this feature along population time *t*_2_, non-oscillating and oscillating contributions can be distinguished. The former describe population decay, while the latter are associated with the evolution of coherent superpositions of states. All of these components can be efficiently and reliably identified using a global analysis methodology based on a complex multiexponential fit.^43,63^

As expected, the non-oscillating evolution of the signal in the investigated time window is not particularly rich, and it is described by a biexponential decay with a first ultrafast component (200 fs) that can be mainly ascribed to the spectral diffusion, and a second much slower contribution (≫2 ps) accounting for relaxation processes characterized by time scales well beyond the investigated 2 ps time window. The 2D decay-associated spectra (2D-DAS) for these two identified time constants are reported in panels e and f of Figure 3. The amplitude distribution of the two components shown in these maps confirms the attribution mentioned above.

The oscillations in the evolution of the 2DES signal can be analyzed by Fourier transforming the rephasing and non-rephasing data sets along *t*_2_. The beating analysis reveals several oscillating components, all corresponding to vibrational modes also detected in Raman spectra. The amplitude distribution of each beating component along the two frequency axes, plotted in the so-called Fourier maps, confirms the vibrational nature of the oscillating signals.^64−66^ Panels g–l of Figure 3 exemplify this analysis for a mode beating at 823 cm^–1^. A further investigation of the sign of the oscillation frequency at specific positions in rephasing and non-rephasing spectra allows an assessment of whether a particular vibrational coherence is evolving in the ground state or the excited state.^59,67^ For the PPh-*ap* monomer, it was determined that the frequency components contributing to the beating pattern in 2D maps can be mainly attributed to ground state vibration (Supporting Information).

While the PPh-*ap* monomer presents all of the features expected for a tetrapyrrole derivative in solution, the 2DES response of the aggregated species reveals a completely different behavior, in terms of both spectral shape and dynamics. The 2D response of the aggregate is summarized in Figure 4, where the positions of the five excitonic states ε*_n_*, identified in the analysis of the linear spectra, are pinpointed by dashed lines. Relevant positions in the 2D maps are quickly identified with coordinates (ε*_n_*, ε*_m_*), where ε*_n_* (ε*_m_*) denotes the frequency associated with exciton state *n* (*m*) on the excitation (emission) axis.

**Figure 4:**
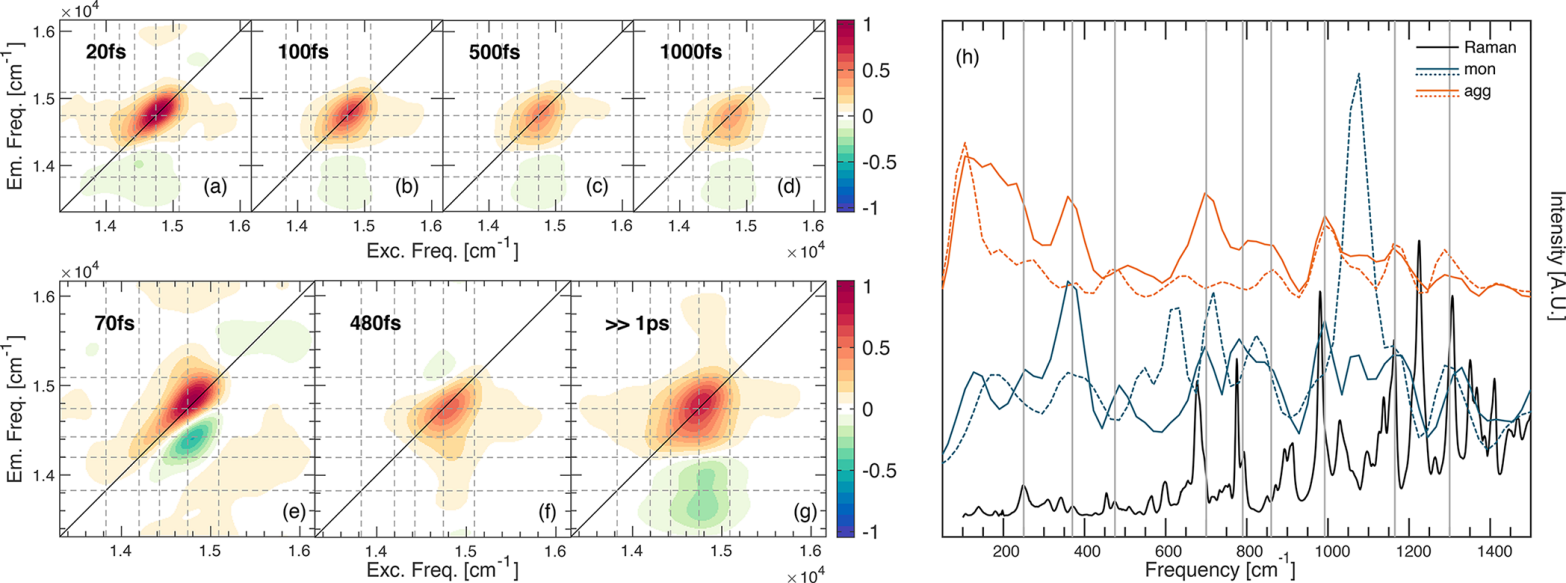
2DES characterization of the PPh-*ap* aggregate. (a–d) Pure absorptive 2DES maps of the aggregate at selected values of population time *t*_2_. Dashed lines pinpoint the positions of transitions ε_*i*_ identified in the linear characterization. (e–g) 2D-DAS retrieved from global fitting analysis of the PPh-*ap* aggregate in the MeOH/H_2_O data set. (h) Power spectra of the beatings in the rephasing (solid line) and non-rephasing (dotted line) data sets for the monomer (blue) and aggregate (orange). Power spectra are obtained by Fourier transforming the 2DES maps along *t*_2_ after integration over the excitation and emission frequencies, and therefore, they capture the main components contributing to the overall beating behavior of the whole 2D maps. The strong signal at ∼1100 cm^–1^ in the non-rephasing spectrum of the monomer is due to the solvent MeOH. The Raman spectrum (excitation at 514 nm) recorded at 77 K on PPh-*ap* powder is also reported for comparison (black line).

Panels a–d of Figure 4 show selected 2D maps at increasing values of population time *t*_2_. The main diagonal peak presents a higher degree of elongation along the diagonal, in agreement with the broader energy distribution also detected in the absorption spectrum. The signal encloses GSB and SE of all of the transitions to the ε*_n_* states identified in the linear spectrum analysis.

A second important feature appearing in the 2D maps is a negative signal below the diagonal, whose intensity is non-negligible even at early population times. This signal clearly indicates the presence of an excited state absorption (ESA) contribution.

The analysis of the dynamics of the 2D map with the global fitting procedure revealed the presence of three non-oscillating decay components, whose associated 2D-DAS are reported in panels e–g of Figure 4. First, a 70 fs component is detected. The associated DAS (Figure 4e) reveal a positive feature elongated on the diagonal and covering all of the bright excitonic states and a negative signal at lower energies.

The amplitude distribution of this DAS cannot be entirely justified with spectral diffusion, which would have given rise to symmetric negative signals above and below the diagonal, as verified for the monomer and other tetrapyrrole compounds.^57,58,61^ Similarly to what was recently observed in different systems,^69−71^ a prominent negative feature below the diagonal witnesses a relaxation from higher- to lower-energy states. Moreover, the distribution of this negative signal along the excitation and emission axes suggests that the relaxation on this time scale mainly involves excitonic states close in energy. Therefore, we attribute the 70 fs time constant to an ultrafast relaxation process of ε*_n_* → ε_*n*–1_ and ε*_n_* → ε*_n_*_–2_ (red arrows in Figure 5).

**Figure 5:**
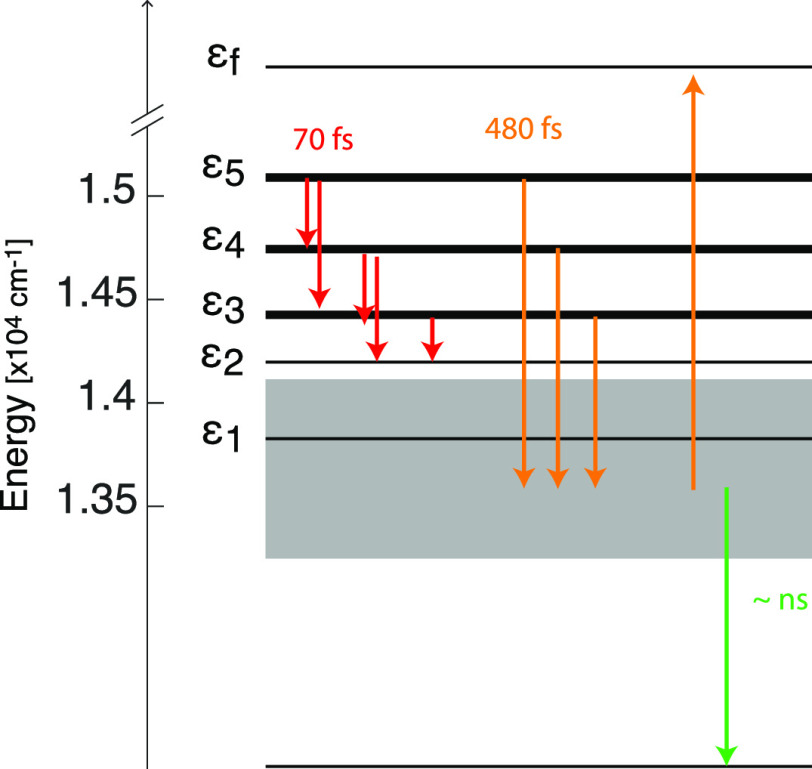
Level scheme and main dynamic processes involved in the excitation energy migration within the Pph-*ap* artificial antenna. Bright states are pinpointed with solid lines, with the thicker lines denoting the brightest states. The gray box identifies a manifold of low-energy dark or almost dark states, whose energy cannot be fully characterized by 2DES. Colored arrows illustrate the main relaxation processes identified in the 2DES measurements.

The second component has a characteristic time of 480 fs. The 2D-DAS (Figure 4f) show a positive amplitude that corresponds the diagonal peak, at coordinates (ε_3_, ε_3_), (ε_4_, ε_4_), and (ε_5_, ε_5_), witnessing a decay of the population of these states with this time constant. A positive amplitude is also recorded in the low cross-peak region at coordinates (ε_4_, ε_1_) and (ε_5_, ε_1_). This last feature is particularly interesting because these coordinates correspond to the position of the negative ESA feature in the 2D maps. A positive amplitude in the exponential fitting of a negative signal means that, overall, the signal is becoming more negative. Physically, this means that the population of the states from which the ESA originates is increasing with a 480 fs time constant. The excitation frequency coordinate of this signal in the 2D maps indicates that the states generating the ESA are populated indirectly after the excitation of states at the ε_4_ and ε_5_ energy; the emission coordinate, instead, suggests that as soon as they become populated, a transition toward higher excited states is promoted. Altogether, this DAS therefore describes a relaxation process that brings the population from bright states ε_3_, ε_4_, and ε_5_ to lower-energy states in hundreds of femtoseconds (orange arrows in Figure 5). Because these states can be populated only indirectly, their energy cannot be precisely identified; however, the fluorescence measures (Figure 1a) suggest that these states may also include ε_1_ and ε_2_ and other lower-energy dark states.

Finally, upon examination of the 2D-DAS relative to the third time components (Figure 4g), a strong negative signal is recorded below the diagonal at ESA coordinates, indicating the decay of population on the low-energy dark states in a time range beyond our experimental window [≫1 ps (green arrow in Figure 5)].

The overall oscillating pattern in the 2DES spectra of the aggregate is very similar to that of the monomer, as shown in the power spectra reported in Figure 4h, obtained by Fourier transforming the 2DES maps along *t*_2_ after integration over the excitation and emission frequencies. The frequency, time behavior, and Fourier maps of the main beating modes correspond with those found in the monomer and attributed to vibrational modes of the tetrapyrrole moiety. However, a more in-depth analysis reveals a few subtle differences. First, a broad signal between 200 and 300 cm^–1^ appears in the aggregate power spectrum, which is absent in the monomer spectrum. This frequency also corresponds to the average energy gap between pairs of adjacent excitonic states. It would be tempting to attribute this signal to electronic coherences, but it was not possible to characterize further the frequency and time properties of this mode because of its quick dephasing. Moreover, it is also known that aggregation can enhance the coupling between low-frequency modes and electronic transitions.^70,72^ In the aggregate, we can surely recognize a more relevant contribution of vibrations in the excited states. This is proven by the similar amplitude of the positive and negative side of the Fourier spectra of beatings (see the Supporting Information).^65,73,74^ Therefore, a partial electronic character should be recognized in these modes, although further investigations are needed to assess the extent to which they might contribute to the overall dynamics and efficiency of the energy transfer process.

In conclusion, we have reported the preparation and photophysical characterization of a biomimetic artificial antenna built by promoting the self-assembly of a suitably designed pigment-peptide conjugate. The pigment, PPh, has been chosen to guarantee intense and spectrally wide absorption bands in the visible range. Instead, the amino acid sequence was designed to promote the formation of a helical secondary structure that can promote self-assembly in water. By exploiting this property, one can reversibly tune the formation of aggregates by modulating only the polarity of the solvent. The spontaneous self-assembly of the amino acid sequences in polar solvents, in turn, constrains the close packing arrangement of the chromophoric side chains, promoting the formation of a dense network of strongly interacting pigments in a reproducible way.

Through MD calculations, we could decipher the fundamental interactions leading to the stabilization of supramolecular assemblies in water and identify the structure of the basic unit (a dimer) constituting the aggregates. These simulations also confirmed the crucial role of the peptide for aggregate formation in different environments.

The proposed system is highly tunable, reproducible, and stable under laser illumination. It guarantees a high level of chemical flexibility due to the possibility of easily functionalizing or replacing the chromophore and peptide moieties. Even more important, this is an example of how it is possible to mimic Nature by exploiting the environment (the solvent polarity, in this case) to tune the photophysics and the light-harvesting capability of complex systems.

The subpicosecond dynamics of energy transport within the manifold of exciton states promoted upon aggregation has been characterized by 2DES and compared with the dynamics in the monomeric form. The main processes and their time scales are schematized in Figure 5. Decades of investigations of the biological antennas suggested that one of the primary dynamics requisites for an efficient antenna is the presence of efficient collector states. Ideally, a collector state should have, on one hand, a lifetime that is sufficiently long to facilitate an efficient transfer to a possible acceptor to which the antenna can be coupled and minimize back transfer. On the other hand, the relaxation dynamics from higher-energy states should be sufficiently fast to minimize deactivation through alternative pathways and losses of excitation energy. In natural light harvesting, this is achieved through the optimization of the distances, orientations, and couplings of the chromophores.

The complexity of the self-assembly process of our antenna does not allow such strict control over the geometrical arrangement of the pigments and their morphology; nevertheless, the study of its ultrafast dynamics revealed the presence of similar behavior. 2DES measures, indeed, confirmed that the excitation energy, initially localized on the high-energy bright states characterized by the greater transition dipole moment, is rapidly transferred in hundreds of femtoseconds to low-energy dark or almost dark states, working in fact as collector states, and characterized by a lifetime much longer than the investigated time window (≫1 ps). The presence of these collector states could be identified only indirectly in the 2DES maps, due to the ESA process in which they are involved. In artificial models of antennas, dark states have been invoked to prevent radiative recombination and increase the transfer efficiency,^30,31,34,75^ but from the experimental point of view, their photophysical and dynamic characterization is often elusive because of their forbidden nature.

The coherent dynamics of the multichromophoric assembly is dominated by ground state vibrational modes, also found in the monomer response. Nevertheless, evidence for low-frequency vibronic contributions has been found, although it was not possible to assess the effective role of these modes in the overall mechanism and dynamics of energy migration, as suggested in other analogous multichromophoric assemblies.^71,76^

The results presented here suggest that the self-assembly of suitably designed pigment-peptide conjugates could be a viable promising approach for preparing artificial light harvesters mimicking the most effective design principles exploited by Nature.

## Supporting Information Available

Supplemental Experimental Procedures (synthesis of the peptide and of the pigment-peptide conjugate, aggregate preparation, and 2DES experimental setup); Supplemental Data Items (Raman spectroscopy measures, fitting of the monomer spectrum, TCSPC, additional R and NR maps, and beating analysis for the monomer and aggregate); and Supplemental Modeling Procedures (PDF)
