# Supplementary material for: Biomimetic Nanoarchitectures for Light Harvesting: Self-Assembly of Pyropheophorbide-Peptide Conjugates
Source: J Phys Chem Lett. 2020 Sep 4;11(19):7972–80. doi: 10.1021/acs.jpclett.0c02138 (PMC8011917; doi:10.1021/acs.jpclett.0c02138)
Supplement: Supplementary file 1 — jz0c02138_si_001.pdf [file jz0c02138_si_001.pdf]

SUPPORTING INFORMATION

Biomimetic Nanoarchitectures for Light-Harvesting: Self-Assembly of pyropheophorbide-Peptide Conjugates

Elena Meneghin[1], Francesca Biscaglia[1], Andrea Volpato[1], Luca Bolzonello[1], Danilo Pedron[1], Elisa Frezza[2], Alberta Ferrarini[1], Marina Gobbo[1], Elisabetta Collini[1]*

[1]*Department of Chemical Sciences, University of Padova, via Marzolo 1, 35131 Padova, Italy*
[2] *Université de Paris, CiTCoM, CNRS, F-75006 Paris, France*

**elisabetta.collini@unipd.it*

S1. Supplemental Experimental Procedures
S1.1. Synthesis of the peptide and of the pigment-peptide conjugate
S1.2. Aggregate preparation
S1.3. 2DES Experimental setup

S2. Supplemental Data Items
S2.1 Vibrational characterization: Raman spectroscopy
S2.2 Fitting of the monomer spectrum
S2.3 TCSPC
S2.4 Additional R and NR maps
S2.5 Beating analysis for the monomer and aggregate

S3. Supplemental Modeling Procedures
S3.1 MD protocols
S3.2 Pigment-peptide distance

S1. Supplemental Experimental Procedures

S1.1 Synthesis of the peptide and of the pigment-peptide conjugate

Materials and Methods. Unless differently specified, all chemicals used in the synthesis are commercial products used without further purification. 9-Fluorenylmethoxycarbonyl (Fmoc)-amino acids and all other chemicals for the solid phase peptide synthesis were purchased from Sigma-Aldrich. H-L-Ala-2-chlorotrityl resin was purchased from Iris Biotech GMBH. Pyropheophorbide *a* (PPh) was bought from Frontier Scientific.
Analytical HPLC separations were carried out on a Dionex Summit dual-gradient HPLC instrument, equipped with four-channel UV-Vis detector, using a Vydac 218TP54 (C18) or a Phenomenex Jupiter (C4) column (250 x 4.6 mm, 5 µm, flow rate at 1.5 mL/min). The mobile phases A (aqueous 0.1% Trifluoroacetic acid (TFA)), B (90% aqueous acetonitrile containing 0.1%TFA) were used for preparing binary gradients. Mass spectral analyses were performed operating in positive mode with ESI technique on a Mariner API-TOF workstation (PerSeptive Biosystems Inc).

Synthesis of the peptide (ap). The artificial peptide (*ap*) sequence (Figure S1) was assembled on an automated Advanced Chemtech 348 Ω starting from H-L-Ala-2-chlorotrityl resin (loading 0.77 mmol/g). The coupling of each residue was carried out in dimethylformamide (DMF) for 60 min, using an excess of the Fmoc-amino acid (4 eq) and in the presence of HATU (4 eq) and N,N,N-ethyldiisopropylamine (8 eq). The Fmoc deprotection step was carried out with 20% piperidine in DMF (5 + 15 min). For control, a small amount of peptide was cleaved from the dry resin by 3 repeated treatments with 30 % hexafluoroisopropanol in CH_2Cl_2 (1 h at room temperature). After each treatment the filtrate was collected and the combined solutions reduced to a small volume. Addition of cold diethyl ether yielded the crude *ap* which was characterized by HPLC and ESI-MS as shown in Table S1.

PPh-peptide conjugate The H-*ap*-resin (0.02 mmol) was swelled in DMF, the solvent was drained off and PPh-OH, diisopropylcarbodiimide, and 1-hydroxybenzotriazole (0.05 mmol each one) in 900 µL of DMF were added to conjugate the peptide to PPh-OH. The reaction mixture was shaken overnight and then filtered to remove the excess of reagents. The resin was repeatedly washed with DMF and CH_2Cl_2 (until the filtrate became colorless) and dried under vacuum. Cleavage from the resin was carried out as described in the previous section and the PPh-*ap* conjugate was characterized as shown in Table S1.

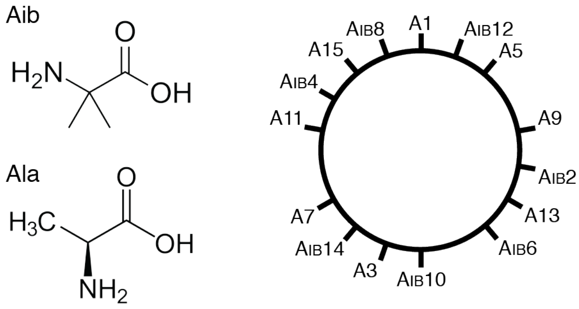

Figure S1. Molecular structure of alanine (Ala) and 2-aminoisobutyric acid (Aib) used in the peptide sequence and helical wheel representation of the Ala-(Aib-Ala)$_7$-OH peptide that highlights its amphiphilicity.

Table S1. HPLC and ESI-MS characterization of *ap* and PPh-*ap*.

Sample	Sequence	Yield (%)	HPLC t_R (min)	Expected Mass $[M+H]^+$	Detected Mass m/z
ap	H-Ala-(Aib-Ala)$_7$-OH	90	16.3 [a]	1182.68	1182.70 $[M+H]^{+1}$, 591.85 $[M+2H]^{+2}$
PPh-*ap*	Pyropheophorbide *a* - Ala-(Aib-Ala)$_7$-OH	98	26.2[b]	1699.34	1699.97 $[M+H]^{+1}$, 849.97 $[M+2H]^{+2}$

[a] Column: C18; elution condition: isocratic 10% B for 3 min; linear gradient 10-90% B for 30 min.

[b] Column: C4; elution condition: isocratic 10% B for 3 min; linear gradient 10-90% B for 30 min

S1.2. Aggregate preparation

In MeOH PPh-*ap* is stable in its monomeric form. According to well-known peptide self-assembly strategies,[1] the aggregation of the PPh-ap conjugate is induced increasing solvent polarity. Five solutions with the same analytic concentration of the conjugate (concentration $4.5 \cdot 10^{-5}\ M$) were prepared, using as solvent different mixtures of MeOH/H_2O with various ratios (v/v). *ap* predominantly retains a α-helix conformation at all the MeOH/H_2O ratios, as demonstrated by the CD spectra recorded in the near UV-region (Figure S2(a)).

The CD spectra in the Vis range (Figure S2(b)) are instead very sensitive to variations of the solvent mixture, since the increase of solvent polarity (at increasingly higher ratio of water) promotes self-assembly of the PPh-ap conjugate. Clear signatures of the typical excitonic behavior are recorded starting from mixtures with a ratio of water higher than 70% (v/v). The increase of the water ratio to values higher than 90% leaded to the precipitation of the sample.

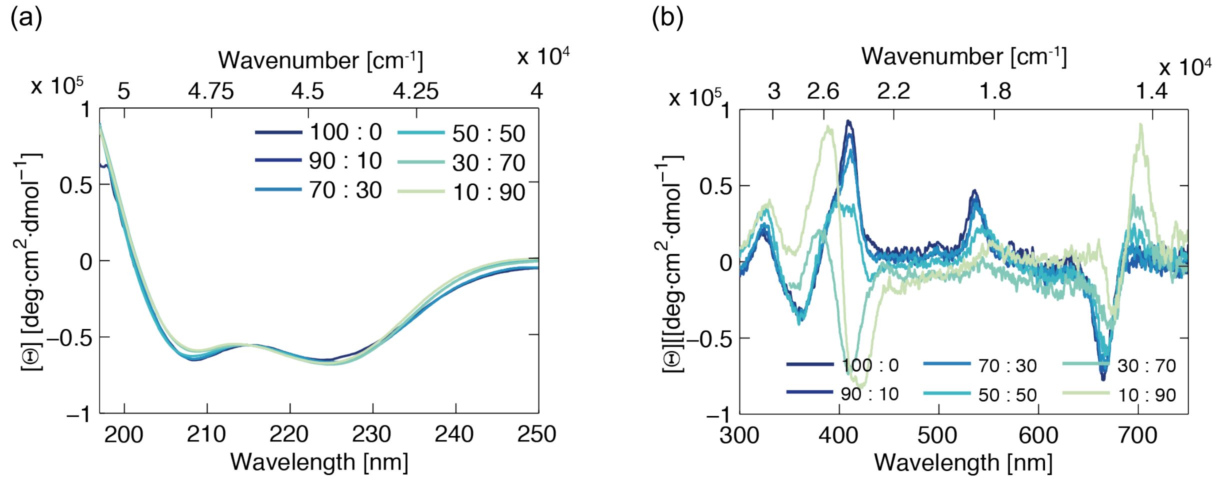

Figure S2. Circular dichroism spectra in (a) the peptide absorption region and (b) in the Vis spectral region, recorded for solutions of PPh-*ap* in mixtures MeOH/H_2O with different ratios. (conc. $4.5 \cdot 10^{-5}\ M$, pathlength 1mm).

S1.3. 2DES Experimental setup

2DES measurements were performed exploiting the setup described in detail in Ref.[2]. Briefly, the output of an 800 nm, 3 kHz Ti:Sapphire laser system (Coherent Libra) is converted into a broad visible pulse in a non-collinear optical amplifier (Light Conversion TOPAS White) and tuned to cover the spectral region of the Q_y transition.

The transform-limited condition for the exciting pulses is achieved at the sample position through a prism compressor coupled with a Dazzler pulse shaper for the fine adjustment. The pulse duration, optimized through FROG measurements is 15 and 10 fs in the experimental conditions used for the measures on monomer and aggregate solution, respectively (Figure S3). The pulses energy at the sample position is reduced to 6 nJ per pulse by a broadband half-waveplate/polarizer system.

The 2DES experiment relies on the passively phase stabilized setup, where the laser output is split into four identical phase-stable beams (three exciting beams and a fourth beam further attenuated of 3 orders of magnitude and used as Local Oscillator, LO) in a BOXCARS geometry using a suitably designed 2D grating. Pairs of 4° CaF_2 wedges control time delays between pulses. One of each pair is translated by stage that regulates the thickness of the medium crossed by the exciting beam and provides a temporal resolution of 0.07 fs. Delay times t_1 (coherence time between first and second exciting pulses), t_2 (population time between second and third exciting pulses) and t_3 (rephasing time between the third exciting pulse and the emitted signal) are thus defined.

The 3D array of data collected during the experiment is Fourier transformed along t_1 and t_3 axes to obtain a dataset of 2D frequency (ω_1) – frequency (ω_3) correlation maps that evolve in t_2. Rephasing and non-rephasing spectra were acquired for population times ranging from 0 up to 1 ps in 5 fs. Each experiment was repeated three times to ensure reproducibility. Steady-state absorption spectrum of the sample was acquired before and after each scan to ensure the sample integrity.

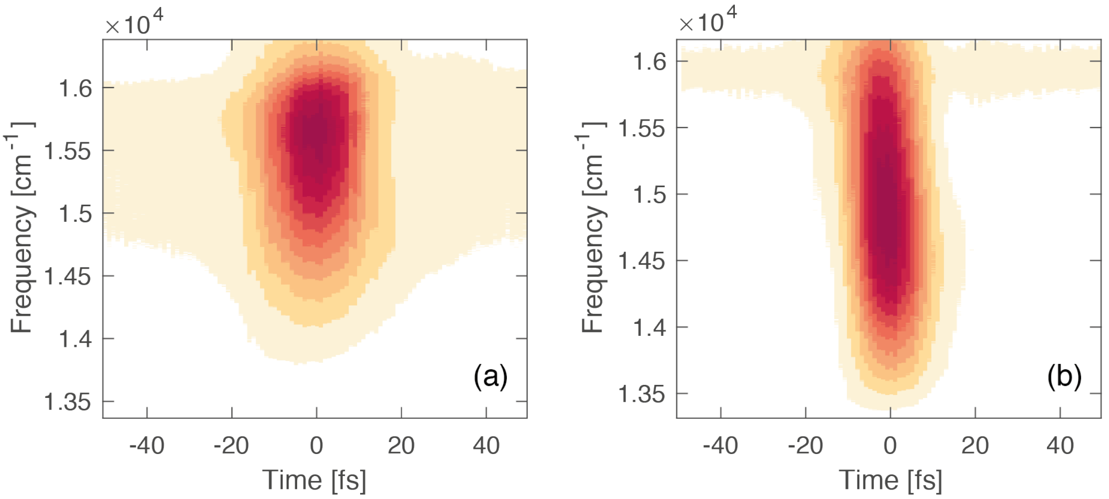

Figure S3. FROG measurements performed at the sample position in a 1mm cuvette filled with solvent in the experimental conditions used for 2DES measures on PPh-ap monomer (a) and aggregate (b). The intensity is normalized to 1 on the maximum. (a) pulse duration (FWHM)=15 fs; (b) pulse duration (FWHM) = 10 fs.

S2. Supplemental Data Items

S2.1 Vibrational characterization: Raman spectroscopy

Raman spectra (Figure S4) were recorded directly on PPh-*ap* powders with a home-built micro-Raman system, based on a Triax-320 ISA spectrograph, equipped with a holographic 1800 g/mm grating and a CCD detector (Spectrum One ISA Instruments). The excitation sources were a Spectra Physics Ar^+ laser operating at 514 nm and an He-Ne laser at 633 nm for non-resonant and resonant conditions, respectively. Appropriate edge filters were used to reduce the stray-light level. An Olympus BX 40 optical microscope equipped with a long working distance 50x/0.50 objective was optically coupled to the spectrograph. The Raman spectra were recorded with an instrumental resolution of about 2 cm^{-1}. To avoid optical damage, the sample was held in a cryostat cell (Linkam Scientific Instruments) at 77 K and the power of the exciting radiation was maintained between 0.2 and 0.4 mW. The value of the Raman shift of the signals appearing in the resonant and non-resonant spectra are reported in the first two columns of Table S2.

Table S2. Raman shift of vibrational modes (in cm^{-1}) recorded on powders of PPh-*ap* at 77 K in resonant (excitation at 633 nm) and non-resonant (excitation at 514 nm) conditions. The superscripts identify intensity properties of the modes: s=strong; m=medium; e=enhanced in resonant conditions. The third column reports the frequency of the beating modes recorded in the Fourier spectrum of coherences (FCS) obtained from 2DES measurements.

Raman		***2DES***
exc@514nm	***exc@633nm***	***FSC***
252	252	250
311	313	
342	344	
371	371	370
455	455	
474	-	480
511	511	
566	566	
-	583	
599	595	
-	639	
677 ^{m}	677	
-	700	700
-	732 ^{e}	
-	741 ^{e}	
755	758	
**775** ^{s}	775	
-	787 ^{e}	785
793	-	
851	-	
870	870 ^{e}	860
892	895	
913 ^{m}	-	
**980** ^{s}	982	995

Raman		***2DES***
exc@514nm	***exc@633nm***	***FSC***
1028 ^{m}	1023	
1050	1050	
-	1071	
1097	1092 ^{e}	
1110	1110	
1127 ^{m}	1126 ^{e}	
1138	1138	
1155	1155	
1164	1167	1170
**1224** ^{s}		
1265		
**1307** ^{s}		1300
**1350** ^{s}		
1367 ^{m}		
1386 ^{m}		
1411 ^{m}		
1447 ^{m}		
1452 ^{m}		
1468 ^{m}		
1498 ^{m}		
1540 ^{m}		
**1555** ^{s}		
**1584** ^{s}		
**1612** ^{s}		

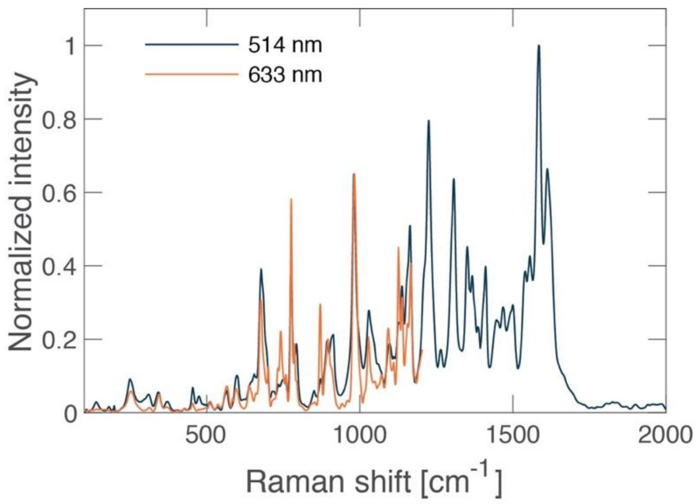

Figure S4. Raman spectra recorded on powder of PPh-*ap* at 77 K in non-resonant (excitation at 514 nm- blue) and resonant (excitation at 633 nm- orange) conditions.

S2.2 Fitting of the monomer and aggregate spectra

The monomer spectrum in the region of the Q_y band has been modeled with a single-transition model function. This approximation is justified considering that in PPh the Q_y - Q_x energy gap is high enough to neglect Q_x contributions in the Q_y spectral window.[3]

The model also includes vibronic couplings. The six vibrational modes more strongly coupled with the electronic transition, basing on the inspection of the resonant Raman spectrum in Figure S4, have been considered. The Huang-Rhys factors of the selected modes for PPh Q_y transition in MeOH are not listed in the literature, therefore their values have been treated as fitting parameters together with the width of the bands, supposed gaussian. The frequency of the modes and the associated HR factors determined with the fit are listed in Table S3.

The fit is in excellent agreement with the experimental trace, as shown in Figure S5(a). The parameters have thus been used to model the lineshape of the single transitions also in the fitting of the aggregate spectrum, under the assumption that the vibronic coupling is not majorly affected by the aggregation process.

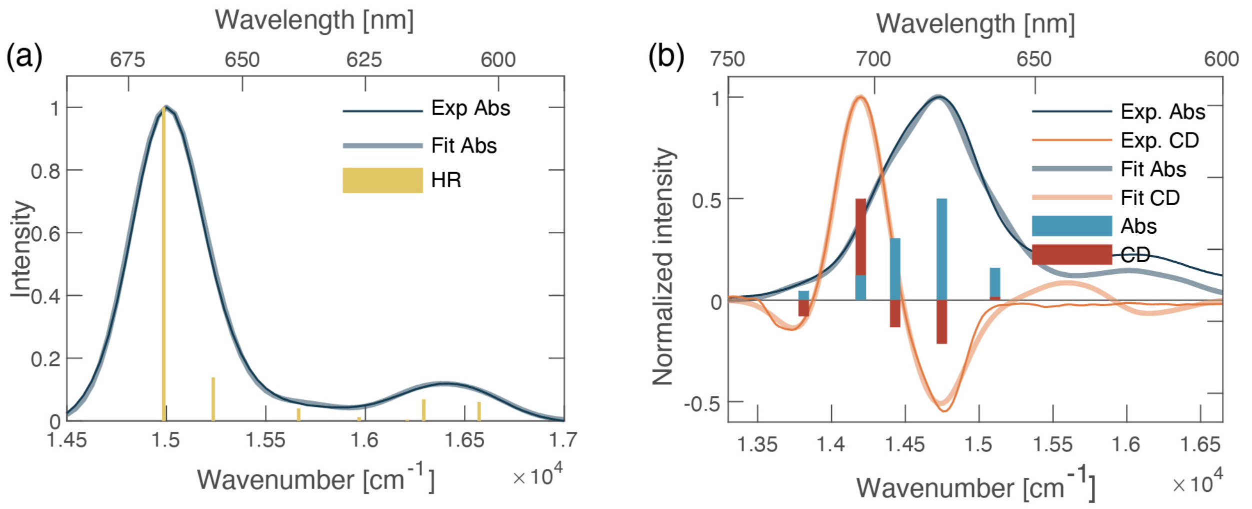

Figure S5. (a) Experimental (blue) and fitted (light blue) traces of the monomer absorption in the Q_y band region. Yellow bars indicate the main vibrational modes included in the fitting model. (b) Experimental (dark thin lines) and global fittings (light thick lines) of the aggregate's absorption (blue) and CD (orange) spectra. The relative amplitudes of the bright transitions are reported as blue and red bars for absorption and CD fitting, respectively.

Table S3. Results of the multi-Gaussian fitting of the monomer absorption spectrum. Frequency (ν_j) and Huang-Rhys factors (S_j) of the vibrational modes mainly coupled to Q_y transition. Fitted values of the site energy and of the Gaussian width are 14987 cm^{-1} and 176 cm^{-1}, respectively.

**ν_j [cm^{-1}]**	249	678	982	1224	1307	1585
**S_j**	0.139	0.040	0.012	0.004	0.069	0.060

The absorption and CD responses of the aggregate have been fitted simultaneously by means of a global fitting procedure (Figure S5(b)) based on the variable projection algorithm, as described in ref.[4]. In this procedure, the frequencies of the transitions have been treated as shared global parameters. To reduce the arbitrariness of a multi-gaussian fitting, for each identified transition, we employed the same lineshape function determined through the fitting of the monomer. The amplitudes (and signs for the CD) of the bands associated to each transition were the only free parameters.

S2.3 TCSPC

Time-resolved fluorescence measures were performed with the time-correlated single photon counting (TCSPC) methodology. The setup is based on a modified configuration of a Jobyn Yvone FluoroMax 3. The standard excitation lamp and detector are replaced by a pulsed nanoled source at 610 nm (Horiba) and a single-photon detector (FluoroHub-B), respectively. The apparatus has a time resolution of about 1.5 ns and the decay was measured in a time window of 100 ns.

Figure S6 shows the fluorescence decay traces of monomer and aggregate solution at room temperature. The monomer decay has a mono-exponential behavior with time constant τ = 7.1 ns. A bi-exponential behavior could be instead retrieved for the aggregate, with time constants τ_1 = 1.7 ns (18%), and τ_2 = 6.3 ns (82%).

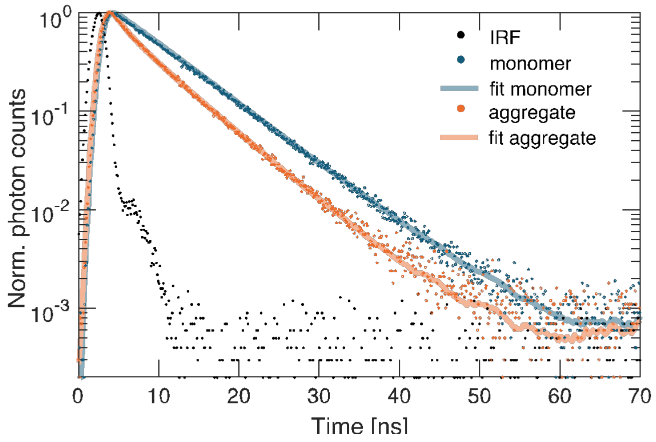

Figure S6. Experimental fluorescence decay traces (dots) and multi-exponential fitting traces (lines).

S2.4. Additional R and NR maps

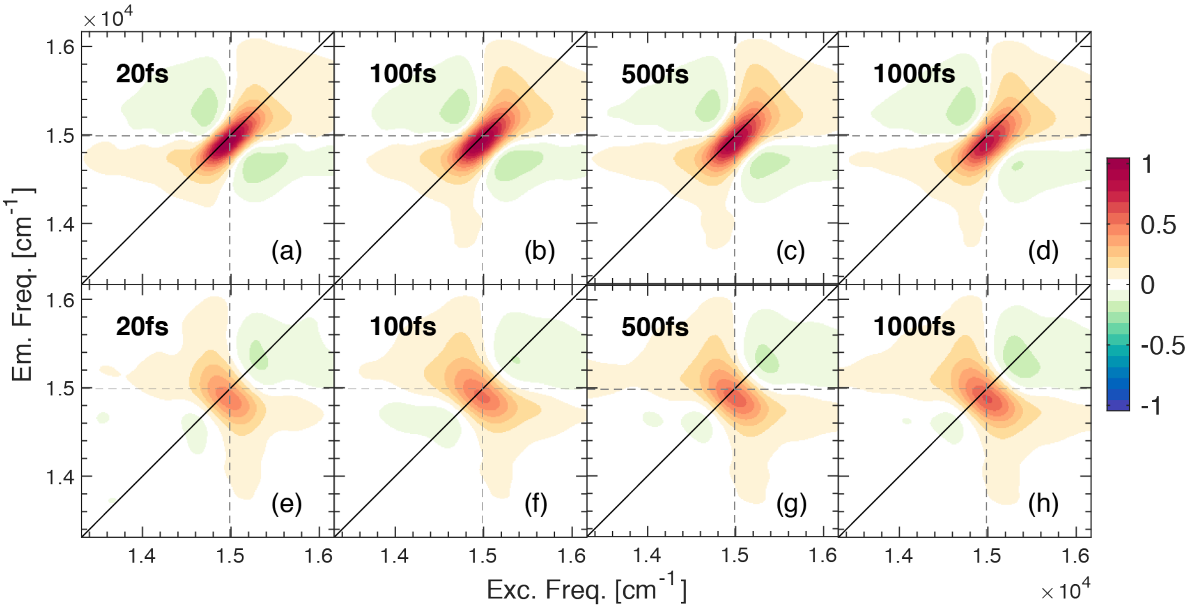

Figure S7. Rephasing (upper row) and non rephasing (lower row) maps of the monomer at selected values of population time t_2.

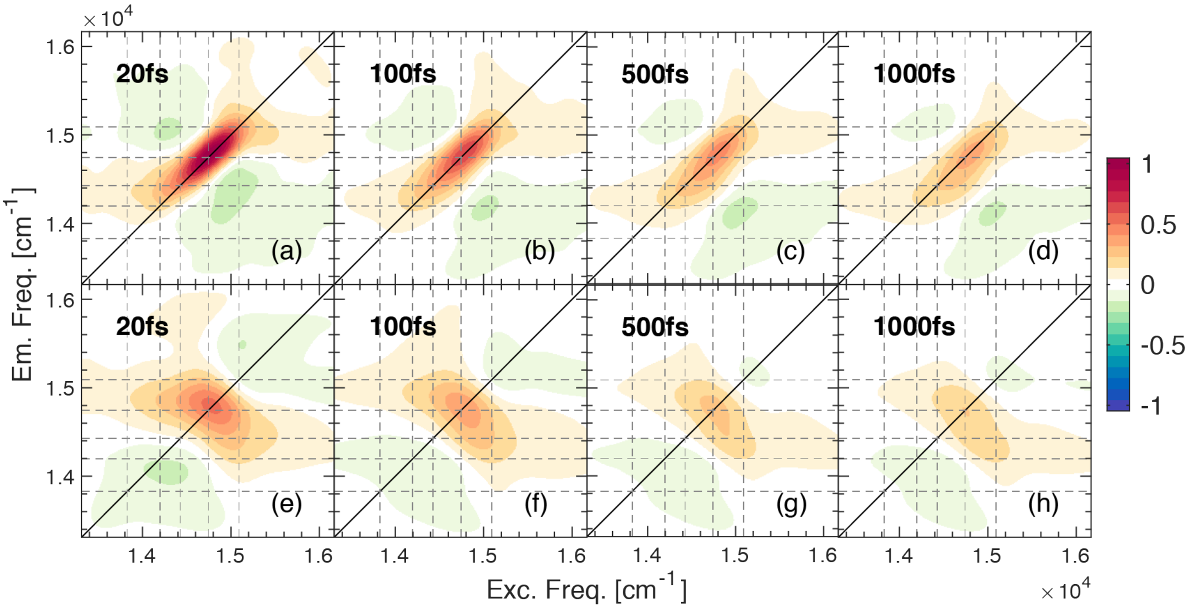

Figure S8. Rephasing (upper row) and non rephasing (lower row) maps of the aggregate at selected values of population time t_2.

S2.5. Beating analysis for the monomer and aggregate

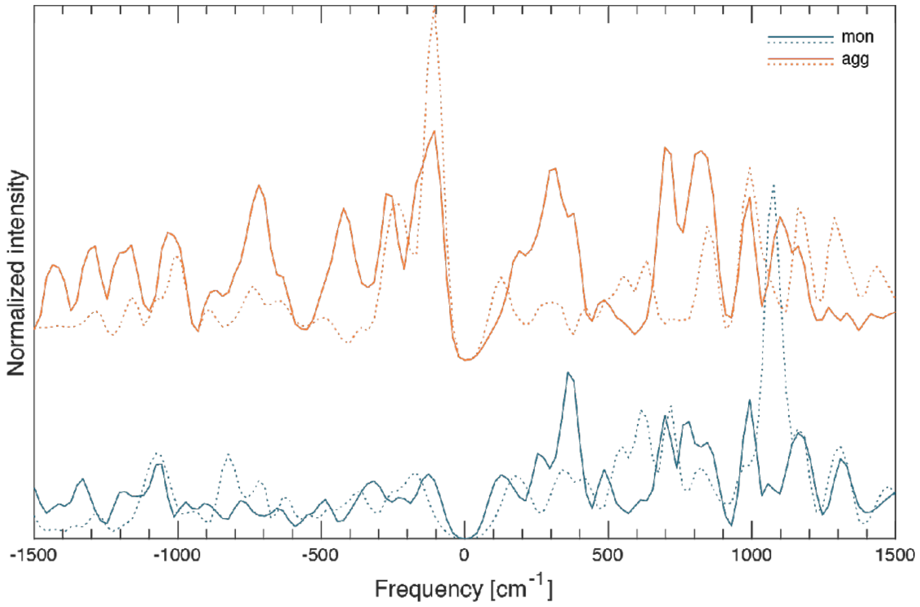

Figure S9. Fourier spectra of the coherent beatings in the rephasing (solid lines) and non rephasing (dashed lines) signal for the monomer (blue) and aggregate (orange). Both negative and positive frequency axis are shown (contributions at about 1000-1100 cm^{-1} are identified as Raman modes of the methanol solvent).

S3. Supplemental Modeling Procedures

S3.1 MD protocols

To perform all atom molecular dynamics (ATMD) simulations, we chose AMBER force field 99SB-ILDN,[5] complemented with force field parameters derived for PPh in Ref.[6] and for AIB in Refs.[7,8]. Additionally, we performed QM calculations to parameterize the atomic charges of the PPh moiety, using the RESP procedure,[9] which is part of the AMBER18 package.[10] The electrostatic potential was obtained by HF calculations with the B3LYP functional and the 6-31G* basis set, whereas atomic coordinates were obtained by DFT with the B3LYP functional and the 6-31G** basis set.[11]

ATMD simulations were carried out for single pigment-peptide conjugates (monomers) and pairs of conjugates (dimers), in explicit water and methanol; the structures were soaked in a cubic box. For methanol, we used the force field proposed by Caldwell and Kollman[12] whereas for water the TIP3P model was used.[13] Periodic boundary conditions (PBC) were used. The cut-off distance for non-bonded interactions was 0.9 Å. Bond constraints were applied to bonds involving H atoms using LINCS algorithm,[14] thus allowing a time step of 2 fs. Electrostatic long-range interactions were evaluated with the particle-mesh Ewald method (PME)[15,16] with a cubic spline approximation. We performed a minimization of 5000 steps: the steepest descent algorithm was used for the first 2500 steps while for the last steps the conjugate gradient algorithm was applied. Then the system was heated up for 60 ps from 1 K to 300 K. In the starting configuration the peptide chain, in helical conformation, pointed away from the PPh moiety and during the simulations the helical conformation remained stable without adding constraints.

For the monomers, the system was equilibrated in water or in methanol for 5 ns and the length of trajectories was 200 ns. For dimers, different starting configurations were selected, with staggered PPh moieties at different angles with respect to each other. Figure S10 shows one of these structures. To avoid stacking of the PPh moieties into H-structures that are not compatible with the UV spectrum, positional restraints were set on the heavy atoms of the pyrrole units (see Figure S11). From an initial value of 1000 kJ mol^{-1}, these were relaxed to 100 kJ mol^{-1} in the initial 1.5 ns, and this value was kept constant in the first part of the production run (200 ns); then, in the subsequent 100 ns the restraints were further reduced to 25 kJ mol^{-1}.

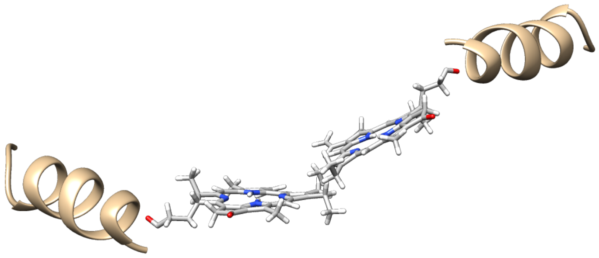

Figure S10. Starting structure of a dimer.

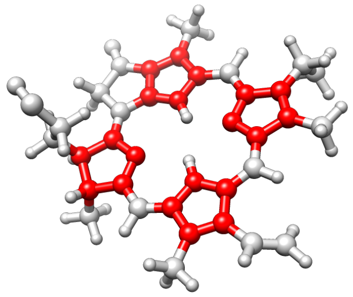

Figure S11. Structure of PPh showing the atoms (red) on which the positional restraints were set in the simulations of dimers.

The simulations were carried out with a stochastic velocity rescale thermostat[17] (τ_T = 0.1 ps) and the Parrinello–Rahman barostat [18] (τ_P = 0.5 ps). A compressibility of 4.5×10^{-5} bar^{-1} was used. All the calculations were performed using Gromacs.[19–21] The trajectories were analyzed using standard and homemade tools.

S3.2 Pigment-peptide distance

Visual inspection of the trajectories shows a clear difference in the behavior of the conjugates in water and in methanol. In the former case, the structures tend to be more globular, with the peptide chains close to the pigment, so reducing the contacts with water. On the contrary, in methanol the peptide chains exhibit larger freedom and can explore a wide range of angles and distances from the pigments.

To characterize this behavior, we have calculated the distance between the center of mass (CoM) of PPh and the CoMs of selected residues in the peptide. Figure S12 shows the results obtained for 7-AIB and 6-ALA, respectively, along ATMD simulations of monomers. In water the distance of the residues from the pigment is short and is characterized by restricted dynamics, whereas in methanol it exhibits significantly larger fluctuations.

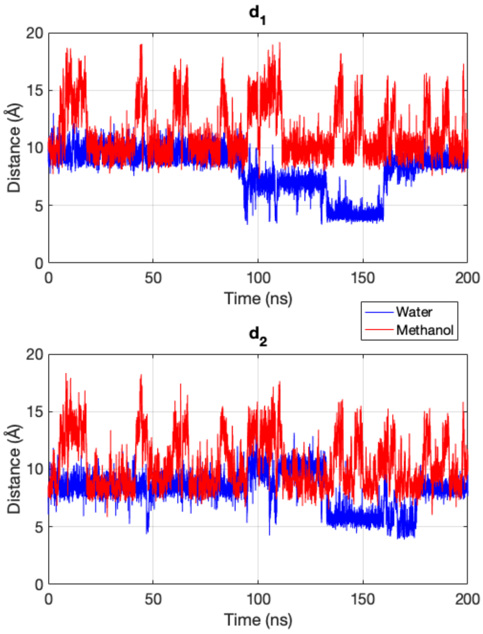

Figure S12. Time series of the distance between the centre of mass (CoM) of PPh and that of 7-AIB (top, d_1) or 6-ALA (bottom, d_2) calculated along ATMD simulations of a monomer in water (blue) and in methanol (red).

Figures S13 and S14 show analogous quantities for the dimers. In particular the former shows the distance between the CoM of 6-ALA in one unit and PPh in the other unit. In water, the peptide chains lie on the PPhs and these distances remain almost constant along the trajectory. On the contrary, in methanol, the peptide chains have higher mobility and stay relatively far apart from the PPhs.

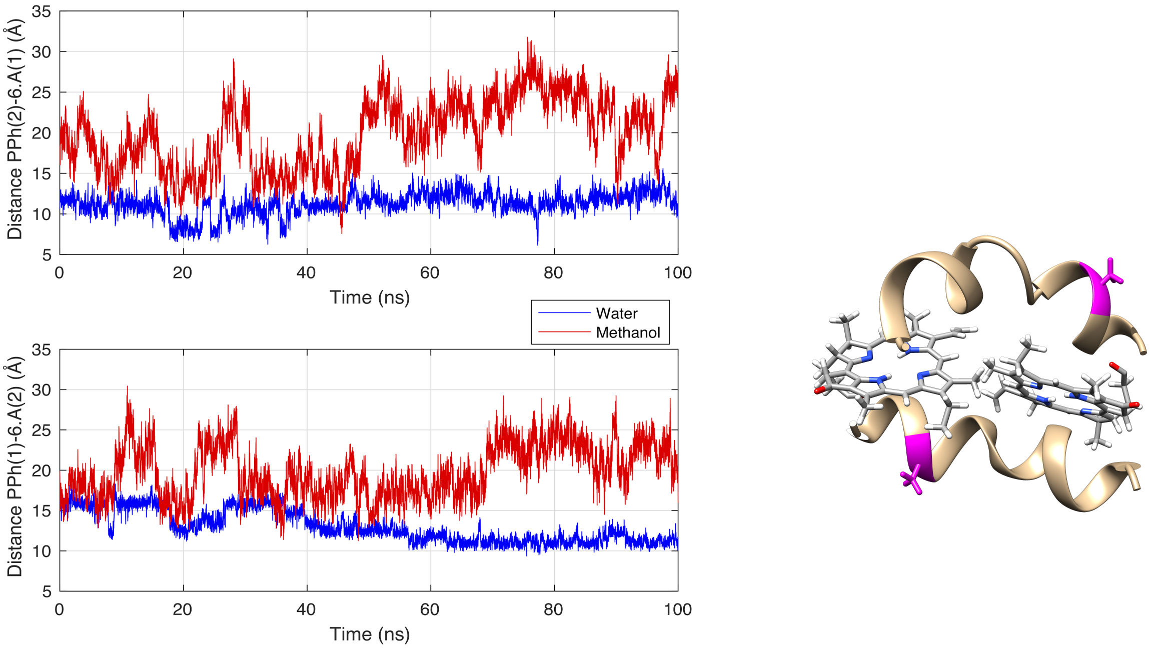

Figure S13. Left: time evolution of the distance between the CoM of each of the PPhs moieties in a dimer and the CoM of the 6-ALA residue linked to the other PPh, calculated along ATMD trajectories in water (blue) and in methanol (red). Right: dimer configuration in a trajectory frame, with the 6-ALA residues highlighted in pink.

Similar results are obtained for the distance between the CoMs of the two PPhs in a dimer, as shown in Figure S14. Starting from a certain distance, enforced by the restraints, a different evolution is exhibited by the dimers in water and in methanol: in the latter the two moieties remain around this distance along the whole trajectory, whereas in the former the PPhs, after a first period, get very close for the remainder of the simulation.

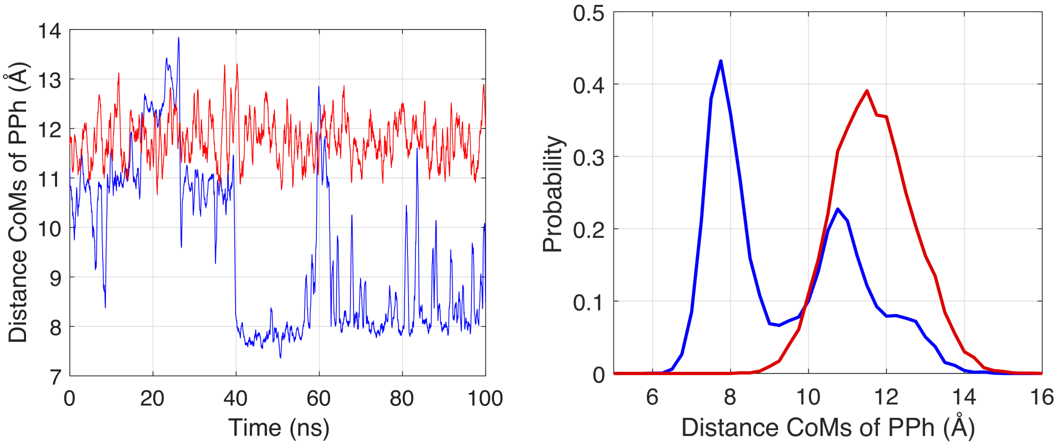

Figure S14. Time series (left) and probability distribution (right) of the distance between the CoMs of PPhs, over ATMD trajectories of a dimer in water (blue) and in methanol (red).

To characterize the fluctuations of the structures, we performed a cluster analysis [22] using the single linkage method as implemented in GROMACS, which includes a structure when its distance to any element of the cluster is less than a given cutoff.[19–21] The distance between two structures is defined as the root mean square deviation after fitting of the structure(s). For the dimer, to improve the quality of the clusters, we considered only the position of the pigments. From each cluster we can extract the structure closest to the center (more representative structure in the cluster). The number of clusters obtained reflects the distinct conformations that are explored along the MD simulations.

References

(1) Kim, S.; Kim, J. H.; Lee, J. S.; Park, C. B. Beta-Sheet-Forming, Self-Assembled Peptide Nanomaterials towards Optical, Energy, and Healthcare Applications. *Small* **2015**, *11*, 3623–3640.

(2) Bolzonello, L.; Volpato, A.; Meneghin, E.; Collini, E. Versatile Setup for High-Quality Rephasing, Non-Rephasing, and Double Quantum 2D Electronic Spectroscopy. *J. Opt. Soc. Am. B* **2017**, *34*, 1223–1233.

(3) Reimers, J. R.; Cai, Z.-L.; Kobayashi, R.; Rätsep, M.; Freiberg, A.; Krausz, E. Assignment of the Q-Bands of the Chlorophylls: Coherence Loss via Qx - Qy Mixing. *Sci. Rep.* **2013**, *3*, 2761.

(4) Volpato, A.; Bolzonello, L.; Meneghin, E.; Collini, E. Global Analysis of Coherence and Population Dynamics in 2D Electronic Spectroscopy. *Opt. Express* **2016**, *24*, 24773–24785.

(5) Lindorff-Larsen, K.; Piana, S.; Palmo, K.; Maragakis, P.; Klepeis, J. L.; Dror, R. O.; Shaw, D. E. Improved Side-Chain Torsion Potentials for the Amber Ff99SB Protein Force Field.

Proteins Struct. Funct. Bioinforma. **2010**, *78*, 1950–1958.
(6) Zhang, L.; Silva, D.-A.; Yan, Y.; Huang, X. Force Field Development for Cofactors in the Photosystem II. *J. Comput. Chem.* **2012**, *33*, 1969–1980.
(7) Grubišić, S.; Brancato, G.; Pedone, A.; Barone, V. Extension of the AMBER Force Field to Cyclic α,α Dialkylated Peptides. *Phys. Chem. Chem. Phys.* **2012**, *14* 15308–15320.
(8) Grubišić, S.; Brancato, G.; Barone, V. An Improved AMBER Force Field for α,α-Dialkylated Peptides: Intrinsic and Solvent-Induced Conformational Preferences of Model Systems. *Phys. Chem. Chem. Phys.* **2013**, *15*, 17395–17407.
(9) Wang, J.; Cieplak, P.; Kollman, P. A. How Well Does a Restrained Electrostatic Potential (RESP) Model Perform in Calculating Conformational Energies of Organic and Biological Molecules? *J. Comput. Chem.* **2000**, *21*, 1049–1074.
(10) Case, D. A.; Ben-Shalom, I. Y.; Brozell, S. R.; Cerutti, D. S.; T.E. Cheatham, I.; Cruzeiro, V. W. D.; Darden, T. A.; Duke, R. E.; Ghoreishi, D.; Gilson, M. K.; et al. AMBER 2018. University of California, San Francisco. 2018.
(11) Frisch, M. J.; Trucks, G. W.; Schlegel, H. B.; Scuseria, G. E.; Robb, M. A.; Cheeseman, J. R.; Scalmani, G.; Barone, V.; Petersson, G. A.; Nakatsuji, H.; et al. Gaussian 16. 2016.
(12) Caldwell, J. W.; Kollman, P. A. Structure and Properties of Neat Liquids Using Nonadditive Molecular Dynamics: Water, Methanol, and N-Methylacetamide. *J. Phys. Chem.* **1995**, *99*, 6208–6219.
(13) Jorgensen, W. L.; Jenson, C. Temperature Dependence of TIP3P, SPC, and TIP4P Water from NPT Monte Carlo Simulations: Seeking Temperatures of Maximum Density. *J. Comput. Chem.* **1998**, *19*, 1179–1186.
(14) Hess, B.; Bekker, H.; Berendsen, H. J. C.; Fraaije, J. G. E. M. LINCS: A Linear Constraint Solver for Molecular Simulations. *J. Comput. Chem.* **1997**, *18*, 1463–1472.
(15) Essmann, U.; Perera, L.; Berkowitz, M. L.; Darden, T.; Lee, H.; Pedersen, L. G. A Smooth Particle Mesh Ewald Method. *J. Chem. Phys.* **1995**, *103*, 8577–8593.
(16) Darden, T.; York, D.; Pedersen, L. Particle Mesh Ewald: An N·log(N) Method for Ewald Sums in Large Systems. *J. Chem. Phys.* **1993**, *98* 10089–10092.
(17) Bussi, G.; Donadio, D.; Parrinello, M. Canonical Sampling through Velocity Rescaling. *J. Chem. Phys.* **2007**, *126*, 14101.
(18) Parrinello, M.; Rahman, A. Polymorphic Transitions in Single Crystals: A New Molecular Dynamics Method. *J. Appl. Phys.* **1981**, *52*, 7182–7190.
(19) Berendsen, H. J. C.; van der Spoel, D.; van Drunen, R. GROMACS: A Message-Passing Parallel Molecular Dynamics Implementation. *Comput. Phys. Commun.* **1995**, *91*, 43–56.
(20) Van Der Spoel, D.; Lindahl, E.; Hess, B.; Groenhof, G.; Mark, A. E.; Berendsen, H. J. C. GROMACS: Fast, Flexible, and Free. *J. Comput. Chem.* **2005**, *26*, 1701–1718.
(21) Hess, B.; Kutzner, C.; van der Spoel, D.; Lindahl, E. GROMACS 4: Algorithms for Highly Efficient, Load-Balanced, and Scalable Molecular Simulation. *J. Chem. Theory Comput.* **2008**, *4*, 435–447.
(22) Everitt, B. S.; Landau, S.; Leese, M.; Stahl, D. *Cluster Analysis, 5th Edition*, 5th ed.; John Wiley & Sons: Chichester, 2011.
